# Strap Performance of N95 Filtering Facepiece Respirators After Multiple Decontamination Cycles

**DOI:** 10.1557/adv.2020.378

**Published:** 2020-12-01

**Authors:** Aaron W. Richardson, Kent C. Hofacre, Patrick H. Keyes, Rachel M. Thurston, John D. Clay

**Affiliations:** Battelle Memorial Institute, USA

## Abstract

The Battelle Critical Care Decontamination System™ (CCDS™) decontaminates N95 filtering facepiece respirators (FFRs) using vapor phase hydrogen peroxide (VPHP) for reuse when there is a critical supply shortage. The Battelle CCDS received an Emergency Use Authorization (EUA) from the US Food and Drug Administration (FDA) in March 2020. This research focused on evaluating the mechanical properties of the straps as an indicator of respirator fit. The objective was to characterize the load generated by the straps following up to 20 don/doff and decontamination cycles in Battelle’s CCDS. In general, the measured loads at 50 and 100% strains after 20 cycles were similar (±15%) to the as-received controls. Qualitatively, reductions in the load may be associated with loss of elasticity in the straps, potentially reducing the ability to obtain a proper fit. However, small changes in strap elasticity may not affect the ability to obtain a proper fit given the potential for variation in strap length and positioning on the head. Regardless, prior to reusing a N95 respirator, it is important to complete a visual inspection to ensure it is not damaged, malformed, or soiled. If so, it is recommended to discard the respirator and use a different one. Similarly, the respirator should be discarded if the wearer cannot obtain a proper fit during the user seal check.

## INTRODUCTION

The severe acute respiratory syndrome coronavirus 2 (SARS-CoV-2) pandemic drove an increase in the demand for N95 filtering facepiece respirators (FFRs). In response to the pandemic, the US Food and Drug Administration (FDA) issued an Emergency Use Authorization (EUA) for the Battelle Critical Care Decontamination System™ (CCDS™) permitting decontamination and reuse of N95 FFRs for up to 20 cycles. The decontamination method should inactivate viruses and bacteria, but not adversely affect filtration performance or fit, and off-gassing of decontamination chemicals should be below the permissible range [1]. EUAs have been issued for several decontamination systems based on vaporous hydrogen peroxide as well as other methods based on supercritical carbon dioxide, ozone, and moist heat [2]. NIOSH research indicates ultraviolet germicidal irradiation (UVGI), vaporous hydrogen peroxide, and moist heat showed the most promise to decontaminate FFRs [1,3,4]. Per Battelle's EUA, the CCDS process is authorized to decontaminate NIOSH-approved N95 FFRs that do not contain cellulose or exhalation valves. This research focused on evaluating the mechanical properties of the straps as an indicator of respirator fit following decontamination using the Battelle CCDS. The objective was to characterize the load generated by the straps from a sampling of ten N95 FFR models following up to 20 decontamination cycles in Battelle's CCDS. The research presented in this paper is specific to strap testing, but Battelle has also demonstrated the aerosol collection efficiency of these 10 models of N95 FFRs is maintained (i.e., ≥95%) after up to 20 VPHP decontamination cycles.

## EXPERIMENTAL DETAILS

The CCDS decontaminates FFRs using vapor phase hydrogen peroxide (VPHP). The VPHP is generated using a commercially available system (Bioquell, Inc., Horsham, PA) and deposits a micro-condensed layer of hydrogen peroxide on the N95 FFR, resulting in inactivation of pathogens, including SARS-CoV-2. Battelle maintains a CCDS for research purposes that is operated using the same protocol as the fielded systems used to decontaminate healthcare N95 FFRs. A total of 10 models of N95 FFRs from 4 different manufacturers were decontaminated up to 20 times in the CCDS research chamber. All FFRs included in the study were compatible with the CCDS process, as they were National Institute for Occupational Safety and Health (NIOSH)-approved N95 FFRs, did not contain cellulose, and did not contain an exhalation valve. Strap materials covered a range of materials including polyisoprene and thermoplastic elastomers based on available information from the manufacturers.

For each model, a batch of FFRs (at least 12) were cycled in the CCDS research chamber. Prior to each VPHP decontamination cycle, each FFR was donned on a manikin head form to simulate a single donning/doffing by a healthcare practitioner. The FFRs were donned by positioning the respirator on the manikin's face and pulling the top and bottom straps over the head form to their proper positions. The FFRs were then immediately doffed and placed in the CCDS chamber for a VPHP decontamination cycle. Typically, three to five FFRs were removed from the batch for strap testing after 5, 10, and 20 VPHP decontamination cycles. The respirators were discarded after strap testing.

The mechanical properties of the strap were tested after 5, 10, and 20 VPHP decontamination cycles. The testing was performed on a Model 5564 Universal Testing System (Instron, Norwood, MA) equipped with a 100 N load cell and pneumatic grips. The upper and lower straps were tested separately, and the results were averaged together for each FFR model. The straps were cut to a length of approximately 13 cm and the grip separation when loaded into the Instron was 10 cm. The strap testing method consisted of three steps: 1) stretch to 200% strain and return to start; 2) stretch to 150% strain and return to start; and 3) stretch to 200% strain. The strain was determined based on crosshead displacement. The load measured during the stretching on the third cycle was reported at 50, 100, 150, and 200% strain. The method was similar to that used by NIOSH to evaluate N95 FFRs after decontamination [5]. In addition to straps from FFRs exposed to the VPHP decontamination process, straps from new, as-received FFRs were also tested as controls for comparison.

## DISCUSSION

Table I summarizes the average measured load after 5, 10, and 20 don/doff and VPHP decontamination cycles for each of the N95 FFRs tested at 50, 100, 150, and 200% strain. Sample size was generally three to five FFRs. The average is provided ±1 standard deviation. The "0" VPHP cycles represents the average of the new, as-received FFR straps used for controls. The delta columns represent the percent change relative to the as-received control. As expected, the load increased with increased strain. The strain experienced during wear depends on the FFR design and the shape/size of the wearer's head. Roberge et al. (2012) measured strains for three different N95 models during wear [6]. The mean strain for the top strap was 52 to 140% and the bottom strap was 22 to 61% across the three models. Thus, a strain of 200% well exceeds that expected to be experienced during wear. Over 80% of the measurements of average load at 50 and 100% strains was within ±10% of the as-received control strap loads.

The effect of the donning/doffing and VPHP decontamination process varied by model, which is reasonable considering the models have different strap construction. In general, the magnitude of changes, if any, observed in the elasticity of the straps increased with increased strain and VPHP decontamination cycles. Increases in the load were observed for Models C, D, and S. An increase in load would be associated with the respirator being pulled tighter to the face. Figure 1 compares the average measured loads as a function of the number of VPHP decontamination cycles and strain for Model D. Straps for Model D contain polyisoprene. The slope of the trendlines increased as the strain increased. Thus, the don/doff and decontamination cycles had more of an effect on the load at the higher strains. For models C, D, and S, the load at the 50 and 100% strains were similar (within 12%) to the controls after up to 20 VPHP cycles.

Decreases in load were observed for Models A, B, E, EE, G, J, and N. Figures 2 and 3 compare average measured loads as functions of VPHP decontamination cycles and strain for Models B and E, respectively, and straps for both are thermoplastic elastomers. In both Figures 2 and 3, the slope of the trendlines were less negative as the strain decreased. A decrease in load has the potential to result in a looser fit of the respirator to the face than a new, as-received FFR. However, at strains likely representative of those during wear (50 and 100% strain similar to the range reported by Roberge et al., 2012), the loads were within 16% of the as-received control. A reduction in force may be associated with a loose-fitting respirator. However, the small change in load observed may not affect fit given variations in strap length and positioning of the straps on the head [7, 8].

Table I. Strap testing results for 10 N95 filtering facepiece respirators.

| N95 | VPHP Cycles | 50% Strain | | 100% Strain | | 150% Strain | | 200% Strain | |
|---|---|---|---|---|---|---|---|---|---|
| | | Max Load[a] (N) | Δ[b] (%) | Max Load (N) | Δ (%) | Max Load (N) | Δ (%) | Max Load (N) | Δ (%) |
| A | 0 | 2.2 ± 0.1 | N/A | 3.5 ± 0.2 | N/A | 5.0 ± 0.3 | N/A | 7.1 ± 0.4 | N/A |
| | 5 | 2.2 ± 0.1 | -1.2 | 3.5 ± 0.2 | -1.3 | 4.9 ± 0.3 | -1.8 | 6.9 ± 0.5 | -2.1 |
| | 10 | 2.1 ± 0.1 | -2.4 | 3.4 ± 0.2 | -3.1 | 4.9 ± 0.3 | -3.5 | 6.8 ± 0.5 | -3.6 |
| | 20 | 1.9 ± 0.1 | -12 | 3.1 ± 0.2 | -12 | 4.4 ± 0.4 | -13 | 6.1 ± 0.6 | -14 |
| B | 0 | 1.3 ± 0.0 | N/A | 2.2 ± 0.1 | N/A | 3.4 ± 0.1 | N/A | 5.8 ± 0.2 | N/A |
| | 5 | 1.3 ± 0.1 | -1.1 | 2.1 ± 0.1 | -3.2 | 3.3 ± 0.2 | -4.3 | 5.7 ± 0.4 | -3.1 |
| | 10 | 1.3 ± 0.0 | -2.4 | 2.1 ± 0.1 | -5.6 | 3.2 ± 0.2 | -7.5 | 5.4 ± 0.4 | -7.2 |
| | 20 | 1.2 ± 0.1 | -9 | 1.9 ± 0.1 | -14 | 2.8 ± 0.2 | -19 | 4.4 ± 0.5 | -24 |
| C | 0 | 1.6 ± 0.1 | N/A | 2.3 ± 0.1 | N/A | 3.0 ± 0.1 | N/A | 3.9 ± 0.1 | N/A |
| | 5 | 1.6 ± 0.1 | 3.0 | 2.4 ± 0.1 | 6.1 | 3.3 ± 0.2 | 8.6 | 4.4 ± 0.3 | 13 |
| | 10 | 1.7 ± 0.1 | 6.9 | 2.6 ± 0.1 | 11 | 3.5 ± 0.1 | 16 | 4.9 ± 0.3 | 25 |
| | 20 | 1.4 ± 0.2 | -11 | 2.3 ± 0.2 | -1.3 | 3.3 ± 0.2 | 10 | 5.2 ± 0.3 | 33 |
| D | 0 | 1.5 ± 0.1 | N/A | 2.2 ± 0.2 | N/A | 2.9 ± 0.2 | N/A | 3.7 ± 0.3 | N/A |
| | 5 | 1.6 ± 0.2 | 6.1 | 2.4 ± 0.2 | 8.6 | 3.2 ± 0.3 | 11 | 4.3 ± 0.5 | 15 |
| | 10 | 1.6 ± 0.1 | 8.7 | 2.5 ± 0.2 | 13 | 3.4 ± 0.3 | 19 | 4.9 ± 0.4 | 31 |
| | 20 | 1.5 ± 0.2 | 0.7 | 2.4 ± 0.3 | 10 | 3.5 ± 0.3 | 23 | 5.7 ± 0.4 | 55 |
| E | 0 | 2.4 ± 0.1 | N/A | 3.5 ± 0.1 | N/A | 4.5 ± 0.1 | N/A | 5.8 ± 0.1 | N/A |
| | 5 | 2.3 ± 0.0 | -2.0 | 3.4 ± 0.1 | -2.6 | 4.4 ± 0.1 | -2.4 | 5.7 ± 0.1 | -1.0 |
| | 10 | 2.2 ± 0.1 | -6.6 | 3.2 ± 0.2 | -8.2 | 4.1 ± 0.3 | -8.3 | 5.3 ± 0.4 | -8.2 |
| | 20 | 2.1 ± 0.1 | -14 | 2.9 ± 0.2 | -16 | 3.7 ± 0.2 | -17 | 4.7 ± 0.3 | -19 |
| EE | 0 | 0.87 ± 0.03 | N/A | 1.4 ± 0.1 | N/A | 1.8 ± 0.1 | N/A | 2.4 ± 0.1 | N/A |
| | 5 | 0.80 ± 0.04 | -7.6 | 1.2 ± 0.0 | -10 | 1.6 ± 0.1 | -13 | 1.9 ± 0.1 | -18 |
| | 10 | 0.81 ± 0.04 | -7.5 | 1.2 ± 0.1 | -10 | 1.6 ± 0.1 | -13 | 1.9 ± 0.1 | -18 |
| | 20 | 0.78 ± 0.04 | -10 | 1.2 ± 0.1 | -13 | 1.5 ± 0.1 | -15 | 1.9 ± 0.1 | -20 |
| G | 0 | 1.0 ± 0.1 | N/A | 1.4 ± 0.1 | N/A | 1.8 ± 0.1 | N/A | 2.3 ± 0.2 | N/A |
| | 5 | 1.0 ± 0.0 | 2.0 | 1.5 ± 0.0 | 6.4 | 2.0 ± 0.1 | 7.6 | 2.5 ± 0.1 | 9.0 |
| | 10 | 1.0 ± 0.0 | 2.8 | 1.5 ± 0.1 | 5.0 | 2.0 ± 0.1 | 7.9 | 2.5 ± 0.1 | 9.3 |
| | 20 | 0.87 ± 0.07 | -13 | 1.3 ± 0.1 | -5.6 | 1.8 ± 0.1 | -1.7 | 2.4 ± 0.2 | 2.4 |
| J | 0 | 1.3 ± 0.1 | N/A | 2.5 ± 0.1 | N/A | 5.8 ± 0.3 | N/A | 39 ± 9 | N/A |
| | 5 | 1.2 ± 0.0 | -1.6 | 2.5 ± 0.1 | -0.8 | 5.9 ± 0.1 | 1.7 | 43 ± 5 | 10 |
| | 10 | 1.2 ± 0.1 | -2.8 | 2.4 ± 0.1 | -3.1 | 5.7 ± 0.2 | -0.2 | 42 ± 5 | 8.3 |
| | 20 | 1.3 ± 0.1 | 5.8 | 2.5 ± 0.1 | 1.9 | 5.5 ± 0.3 | -4.7 | 30 ± 7 | -24 |
| N | 0 | 0.80 ± 0.08 | N/A | 1.3 ± 0.1 | N/A | 1.7 ± 0.1 | N/A | 2.3 ± 0.2 | N/A |
| | 5 | 0.80 ± 0.06 | -0.5 | 1.3 ± 0.1 | -1.9 | 1.6 ± 0.1 | -4.2 | 2.1 ± 0.2 | -9.0 |
| | 10 | 0.79 ± 0.06 | -1.8 | 1.3 ± 0.1 | -2.3 | 1.6 ± 0.1 | -4.3 | 2.1 ± 0.1 | -8.5 |
| | 20 | 0.76 ± 0.07 | -5.3 | 1.2 ± 0.1 | -7.1 | 1.5 ± 0.2 | -10 | 1.9 ± 0.2 | -16 |
| S | 0 | 2.4 ± 0.1 | N/A | 4.3 ± 0.1 | N/A | 6.1 ± 0.1 | N/A | 9.0 ± 0 | N/A |
| | 5 | 2.5 ± 0.1 | 4.8 | 4.6 ± 0.2 | 7.7 | 7.1 ± 0.7 | 16 | 12 ± 2 | 31 |
| | 10 | 2.5 ± 0.1 | 5.7 | 4.5 ± 0.2 | 5.7 | 7.0 ± 0.4 | 14 | 12 ± 1 | 32 |
| | 20 | 2.7 ± 0.1 | 12 | 4.5 ± 0.1 | 3.8 | 6.6 ± 0.4 | 7.5 | 11 ± 1 | 26 |

[a] Average ±1 standard deviation.

[b] Percent change relative to as-received control.

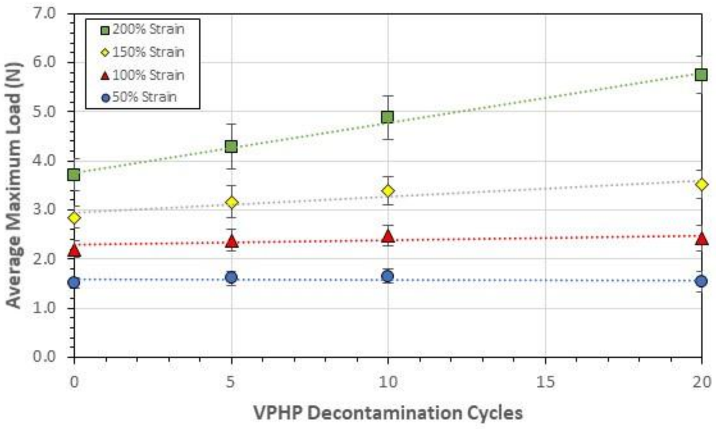

Figure 1. Average measured load of Model D N95 FFR at 50, 100, 150, and 200% strain.

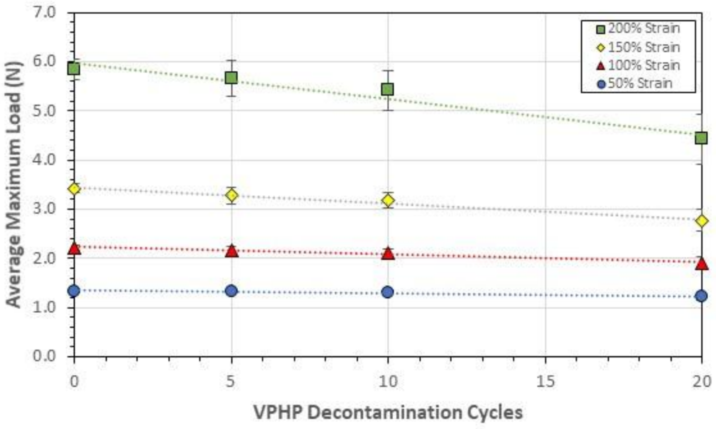

Figure 2. Average measured load of Model B N95 FFR at 50, 100, 150, and 200% strain.

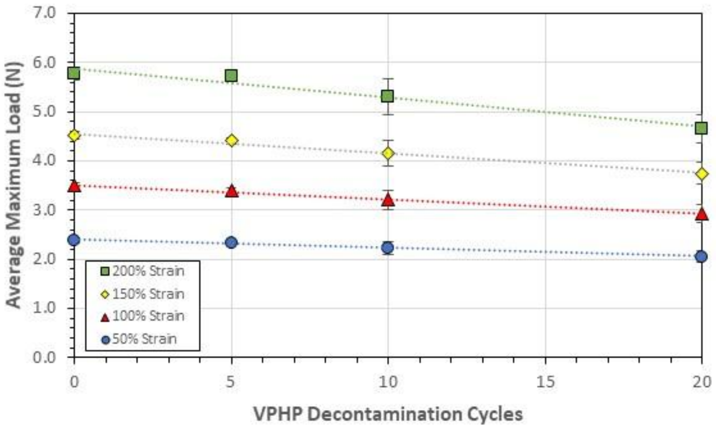

Figure 3. Average measured load of Model E N95 FFR at 50, 100, 150, and 200% strain.

For Model E, additional testing was performed to determine whether the reduction in load was attributed to the repeated donning/doffing or the VPHP process. Model E FFRs were donned/doffed on the head form up to 10 times and not exposed to the VPHP decontamination cycle. In addition, Model E FFRs were cycled through the VPHP decontamination process without any donning/doffing on a head form. The measured loads at 100% strain are compared in Figure 4. The load of the "don/doff only" straps were 14% lower than the as-received control after 10 don/doff cycles (data not collected after 20 don/doff cycles due to limited availability of FFRs). In comparison, the load of the "VPHP only" straps were within 3% of the as-received control even after 20 VPHP decontamination cycles. Thus, the donning/doffing process results in the reduction of the stress/load and not exposure to the VPHP decontamination process. In comparison, similar testing on Model D demonstrated that the VPHP cycling process led to the observed increase in strap force (data not shown). The impact, if any, of the VPHP decontamination process is dependent on the strap design and materials of construction.

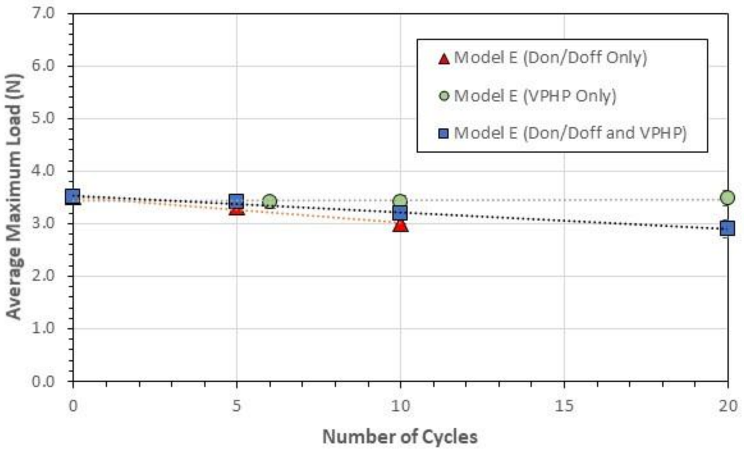

Figure 4. Comparison of average measured load of Model E N95 FFR at 100% strain for the don/doff only, VPHP only, and combination don/doff and VPHP.

## CONCLUSIONS

Although the preference is always to wear a new FFR when available, a proven decontamination technique to allow reuse of N95 FFRs ensures that healthcare practitioners will remain protected when there is a critical supply shortage. This research evaluated changes in the elasticity of straps from 10 different models of N95 FFRs after repeated don/doff and VPHP decontamination cycles (up to 20). The mechanical properties of the straps were characterized as an indicator of respirator fit. In general, the measured loads at 50 and 100% strains after 20 cycles were similar (±15%) to the as-received controls. Larger changes were observed at strains up to 200%, but this strain is likely higher than that experienced during wear.

The relationship between strap properties and respirator fit has not been well-characterized in the literature. Qualitatively, reductions in the load may be associated with loss of elasticity in the straps, potentially reducing the ability to obtain a proper fit. However, small changes in strap elasticity may not affect the ability to obtain a proper fit given the potential for variation in strap length and positioning on the head [7, 8]. Further research is recommended to characterize the relationship between strap properties and respirator fit.

It is recommended to follow CDC NIOSH guidance when considering decontamination of FFRs for reuse [1]. Decontamination and subsequent reuse of FFRs should only be practiced where FFR shortages exist. Healthcare workers should receive training on FFR reuse. Fit performance during limited reuse, including decontaminated FFRs, should be monitored by the respirator program manager or appropriate safety personnel. If respirator supplies are limited, OSHA recommends to re-assess engineering controls, work practices, and administrative controls to identify any changes that can decrease the need for respirators [4].

The number of times a respirator can be reused is limited by several factors, regardless of whether the respirator is decontaminated between uses. Prior to reuse, it is important to visually inspect the FFR and to perform a user seal check. If the FFR is damaged or soiled or a proper seal cannot be established, it is recommended to discard the respirator and use a different one.

## ACKNOWLEDGEMENTS

This research was funded by the Defense Logistics Agency (DLA).
